# The vertebrate makorin ubiquitin ligase gene family has been shaped by large-scale duplication and retroposition from an ancestral gonad-specific, maternal-effect gene

**DOI:** 10.1186/1471-2164-11-721

**Published:** 2010-12-20

**Authors:** Astrid Böhne, Amandine Darras, Helena D'Cotta, Jean-Francois Baroiller, Delphine Galiana-Arnoux, Jean-Nicolas Volff

**Affiliations:** 1 Institut de Génomique Fonctionnelle de Lyon, Université de Lyon, Université Lyon 1, CNRS, INRA, Ecole Normale Supérieure de Lyon, Lyon, France; 2 CIRAD, Upr20, Dept. Persyst, Campus International de Baillarguet, Montpellier, France

## Abstract

**Background:**

Members of the makorin *(mkrn) *gene family encode RING/C3H zinc finger proteins with U3 ubiquitin ligase activity. Although these proteins have been described in a variety of eukaryotes such as plants, fungi, invertebrates and vertebrates including human, almost nothing is known about their structural and functional evolution.

**Results:**

Via partial sequencing of a testis cDNA library from the poeciliid fish *Xiphophorus maculatus*, we have identified a new member of the *makorin *gene family, that we called *mkrn4*. In addition to the already described *mkrn1 *and *mkrn2*, *mkrn4 *is the third example of a makorin gene present in both tetrapods and ray-finned fish. However, this gene was not detected in mouse and rat, suggesting its loss in the lineage leading to rodent murids. *Mkrn2 *and *mkrn4 *are located in large ancient duplicated regions in tetrapod and fish genomes, suggesting the possible involvement of ancestral vertebrate-specific genome duplication in the formation of these genes. Intriguingly, many *mkrn1 *and *mkrn2 *intronless retrocopies have been detected in mammals but not in other vertebrates, most of them corresponding to pseudogenes. The nature and number of zinc fingers were found to be conserved in Mkrn1 and Mkrn2 but much more variable in Mkrn4, with lineage-specific differences. RT-qPCR analysis demonstrated a highly gonad-biased expression pattern for *makorin *genes in medaka and zebrafish (ray-finned fishes) and amphibians, but a strong relaxation of this specificity in birds and mammals. All three *mkrn *genes were maternally expressed before zygotic genome activation in both medaka and zebrafish early embryos.

**Conclusion:**

Our analysis demonstrates that the *makorin *gene family has evolved through large-scale duplication and subsequent lineage-specific retroposition-mediated duplications in vertebrates. From the three major vertebrate *mkrn *genes, *mkrn4 *shows the highest evolutionary dynamics, with lineage-specific loss of zinc fingers and even complete gene elimination from certain groups of vertebrates. Comparative expression analysis strongly suggests that the ancestral E3 ubiquitin ligase function of the single copy *mkrn *gene before duplication in vertebrates was gonad-specific, with maternal expression in early embryos.

## Background

**Figure 1:**
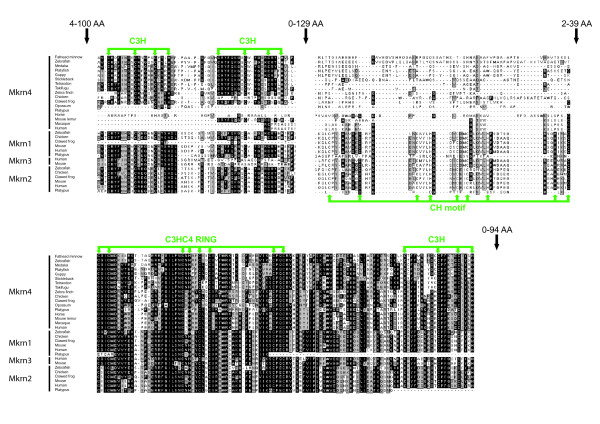
**Zinc finger domains in Mkrn4 and other vertebrate Makorin proteins**. Green arrows show conserved cysteine and histidine residues in zinc finger motifs. Minimal/maximal numbers of amino-acid residues between motifs are indicated over black arrows. Black columns indicate identical amino-acids, grey columns similar residues.

Despite their presence in organisms as diverse as fungi, plants and animals, the functions and evolution of Makorin (Mkrn) proteins in eukaryotes remain poorly understood. Makorins are zinc finger proteins with a typical C3HC4 motif called the RING domain. This protein-protein interaction motif is found in most E3 ubiquitin ligases, a category of enzymes mediating the transfer of ubiquitin from an E2 ubiquitin-conjugating enzyme to target protein substrates. The RING domain is responsible for the ubiquitin ligase activity, leading to mono-ubiquitination and/or to synthesis of poly-ubiquitin chains on lysine residues (for review [1]). Accordingly, some Makorin proteins work as E3 ubiquitin ligases [2]. In Makorin proteins, the RING domain is associated with typical arrays of one to four C3H domains, a type of zinc finger found in a variety of ribonucleoproteins [3]. Another motif rich in Cys and His residues, with so far unknown function, is also generally present in Mkrn proteins [4](Figure 1).

Three functional *mkrn *genes, *mkrn1*, *2 *and *3*, have been described so far in vertebrates, with *mkrn3 *being specific to therian mammals. Makorin1 has been well studied in mammals. Its described functions are linked to E3 ubiquitin ligase activity, for example ubiquitination-mediated degradation of capsid proteins as a defence mechanism against virus infection [5]. Mkrn1 is also involved in the control of cell cycle arrest and apoptosis through ubiquitination and proteasome-dependent degradation of proteins p53 and p21 [6]. Mkrn1 targets human telomerase catalytic subunit (hTERT) for proteasome processing during differentiation or cell cycle arrest [7]. Makorin1 might also have transcriptional activity and regulates RNA polymerase II-dependent transcription independently from E3 ligase activity, with either negative or positive effects on gene expression [8]. Changes in *mkrn1 *expression have been associated with different types of cancer and other diseases, as observed for other RING proteins with E3 ligase activity [9-12].

A close relative to *mkrn1 *is *mkrn3 *(aka *Znf127 *in human and mouse), which has been detected only in therian mammals. *Mkrn3 *has been first identified in the Prader-Willi syndrome critical region on 15q11.2 in the human genome [13,14], but with no obvious role in the disease [15]. No function has been assigned so far to Mkrn3. *Mkrn3 *in fact corresponds to an intronless retrocopy of *mkrn1 *generated through reverse transcription of an *mkrn1 *mRNA molecule. The formation of such retrogenes is catalyzed by the reverse transcriptase encoded by autonomous retrotransposable elements. Several other retrocopies of *mkrn1 *have been identified in mammalian genomes, most of them probably corresponding to pseudogenes. The possible involvement of such pseudogenes in the regulation of the founding source gene *mkrn1 *has been proposed and debated [8,13,14,16].

The third functional member of the makorin family described in vertebrates is *mkrn2*. This gene partially overlaps with the *raf1 *proto-oncogene in antisense transcriptional orientation [17]. Functional data have been only published for the clawed frog *Xenopus laevis*, where Mkrn2 is a neurogenesis inhibitor acting upstream of glycogen synthase kinase-3beta (GSK-3beta) in the phosphatidylinositol 3-kinase/Akt pathway. The third C3H zinc finger, the Cys-His motif as well as the RING zinc finger are necessary for this anti-neurogenic activity [18,19].

During the screening of a testis cDNA library from the platyfish *Xiphophorus maculatus *(poeciliid), we have identified a gene with gonad-specific expression representing a new divergent member of the makorin gene family in vertebrates, which was named *mkrn4*. Comparative analyses in different species revealed the evolutionary dynamics of this family of RING finger proteins, suggesting an ancestral gonad-specific function and maternal embryonic expression before duplication in vertebrates.

## Results and discussion

### *Mkrn4 *is a new member of the vertebrate makorin gene family

In order to identify new potential gonad-specific transcription factors or regulatory proteins expressed in fish gonads, we randomly sequenced clones from a testis cDNA library of the platyfish *Xiphophorus maculatus. In silico *screening for sequences containing zinc finger domains revealed a clone with similarity to uncharacterized members of the makorin gene family [20] (Genbank accession number HQ377193). Since three functional *mkrn *genes have been described so far in vertebrates, the newly identified gene was called makorin4 (*mkrn4*).

**Figure 2:**
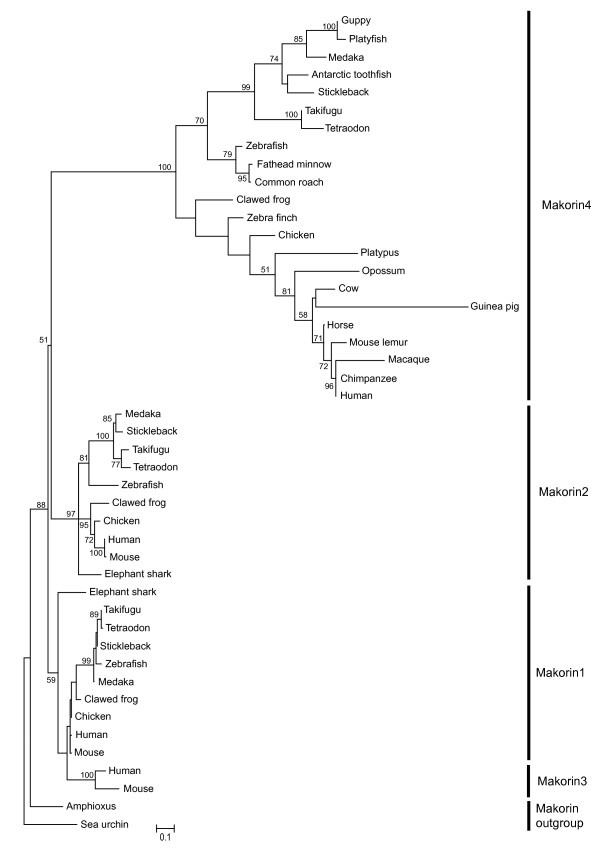
**Molecular phylogeny of the Makorin family**. The tree was constructed using maximum likelihood analysis with 1000 bootstrap replicates on a 131 amino acid MUSCLE alignment of the RING domain and the C-terminal C3H zinc finger. The tree was rooted using the sequence of the sea urchin. Accession numbers of proteins used in the alignment are given in Additional file 1.

We could identify sequences orthologous to platyfish *mkrn4 *in other fish species (Figures 1 and 2). All fully sequenced fish genomes including zebrafish (*Danio rerio*), medaka (*Oryzias latipes *[21], green spotted pufferfish (*Tetraodon nigroviridis*),[22], fugu (*Takifugu rubripes*) [23] and three-spined stickleback (*Gasterosteus aculeatus*) contained *mkrn4 *as a single copy gene. *Mkrn4 *ESTs were also identified in databases for other fish species without available genome draft. *Mkrn4 *is present in birds (on Gga26 in chicken) and amphibians as well as in most mammals, including human (Hsa6q14.1, where it was annotated as a pseudogene, see below). Mkrn4 sequences form a clear monophyletic group within the Makorin molecular phylogeny (Figure 2).

Surprisingly, analysis of genome sequence drafts and EST data failed to detect *mkrn4 *in two murid rodents, the mouse *Mus musculus *and rat *Rattus norvegicus*, even after targeted scrutiny of chromosomal segments syntenic to *mkrn4*-containing loci from other species (on Mmu17 in mouse and Rno20 in rat). Partial *mkrn4 *sequences were found in other rodents such as Ord's kangaroo rat (*Dipodomys ordii)*, thirteen-lined ground squirrel (*Spermophilus tridecemlineatus) *and Guinea pig (*Cavia porcellus*; for this species a deduced sequence could be included into the Mkrn phylogeny), as well as in the American pika *Ochotona princeps *(lagomorph). No *mkrn4*-related sequences were identified in the genome draft of another lagomorph, the rabbit *Oryctolagus cuniculus*, and in the northern tree shrew *Tupaia belangeri *(order Scandentia). For all these species, no *mkrn4 *EST sequence was present in databases. Hence, these results suggest that the *mkrn4 *gene has been lost in murid rodents (mouse and rat). In some other rodent or lagomorph species, *mkrn4 *may be also on the way of degradation (as suggested by long branch for Guinea pig sequence; Figure 2) or even might have been already eliminated, but genomic sequences of better quality will be required to confirm this hypothesis.

We noticed that *mkrn4 *was annotated as a pseudogene in the current Ensembl human genome release, under the name of *Mkrnp2 *[24]. Very similar orthologous sequences were identified in syntenic regions of the genome of chimpanzee (chr. 6, 99% nucleotide identity with the human sequence in the coding region), orangutan (chr. 6, 99% nucleotide identity) and macaque (chr. 4, 96% nucleotide identity), a degree of conservation surprising for a pseudogene evolving under neutral selection. Only gorilla did not show any *mkrn4 *sequence, despite the presence of a large syntenic region on chromosome 6 including several genes surrounding *mkrn4 *in other primates. However, the draft sequence of this region contains many gaps, especially at the position where *mkrn4/mkrnp2 *would be expected. Hence, *mkrn4 *might be absent from draft but present in the genome of the gorilla.

In human, *mkrnp2 *was annotated as pseudogene due to a premature stop codon at position 53 in the Ensembl transcript. We suggest that the translation start codon was wrongly annotated in the Ensembl prediction. A start codon in frame +3, using the ATG at position 54 of the predicted transcript, leads to a full length protein without any further interruption of the open reading frame. This ATG is conserved between primate sequences and annotated as the *bona fide *start codon in GenBank entry EAX03829.1 used in sequence alignments and molecular phylogenies (Figures 1 and 2). Primate sequences were reannotated accordingly (Figure 1).

Using primate sequences as query against EST databases in BLAST analysis, no evidence of transcription could be found. We then compared the rate of non-synonymous (Ka) to the rate of synonymous substitutions (Ks) between *mkrn *sequences in primates (human, chimpanzee, orangutan and macaque) and other vertebrates. The average Ka/Ks value obtained between primate *mkrn4 *sequences was 0.12, indicating an excess of synonymous vs. non-synonymous substitutions. This value was similar to those obtained between primate *mkrn1 *(0.16) and *mkrn2 *(0.11). The average Ka/Ks ratio for *mkrn4 *between fish sequences (medaka, platyfish and guppy) was 0.27. Taken together, these observations suggest that *mkrn4 *evolved under purifying selection in primates and fish, as observed for other *makorin *genes. *Mkrn4 *might be a functional protein-coding gene in primates but expressed at a very low level, in a particular type of cells or under very specific conditions. Alternatively, *mkrn4 *might have lost its expression potential after initial functionality in different primate species.

No *mkrn4 *sequence was found in the genome of more divergent species, including the elephant shark *Callorhinchus milii *(Chondrichthyes *aka *cartilaginous fish, a divergent jawed vertebrate) [25] and the invertebrate chordates *Branchiostoma floridae *(amphioxus) and *Ciona intestinalis/savignyi *(sea squirts).

### Large-scale duplication and the evolution of the makorin gene family

*Mkrn1*, *mkrn2 *and *mkrn4 *are all present in both ray-finned fish and tetrapod lineages, indicating that these genes have been formed through gene duplications at least 450 million years ago early in the vertebrate lineage. *Mkrn1 *and *mkrn2 *but not *mkrn4 *were detected in the genome of elephant shark *Callorhinchus milii*, a cartilaginous fish. In contrast, we could identify only one *makorin *gene in amphioxus and sea squirts (non-vertebrate chordates), as well as in other more divergent non-vertebrate species including yeast and rice. In the fly *Drosophila melanogaster*, four *makorin *genes were identified, but three of them clearly corresponded to retrocopies of the only *bona fide *intron-containing *mkrn *gene. Hence, the *mkrn *gene family observed in vertebrates is probably the result of two events of gene duplication having taken place between the emergence of vertebrates and the divergence between tetrapods and ray-finned fish. At least the duplication having led to *mkrn1 *and *mkrn2 *occurred before the split between cartilaginous and bony fish.

In order to know if gene duplications at the origin of the *mkrn *family might be linked to rounds of whole genome duplications having occurred at the basis of the vertebrate lineage [26-29], we tested if *mkrn *genes might be included in large ancient duplicated regions called paralogons having resulted from such events.

**Figure 3:**
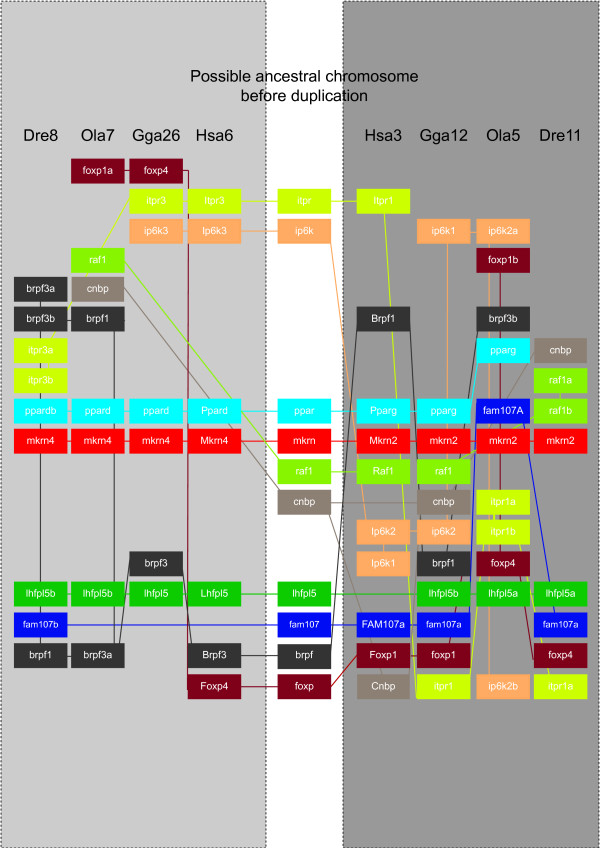
***Mkrn2 *and *mkrn4 *are included in large paralogons in bony vertebrates**. Genes are indicated in colored boxes. Colored lines connect orthologs on one side with paralogs on the other side of the figure. Each grey panel contains a set of orthologous syntenic regions, the putative ancestral region before duplication is shown in the central white panel. Dre: *Danio rerio *(zebrafish); Gga: *Gallus gallus *(chicken); Hsa: *Homo sapiens *(human); Ola: *Oryzias latipes *(medaka fish). Accession numbers of genes presented in this figure are listed in Additional file 2.

On human chromosome 3, *mkrn2 *and the *raf1 *proto-oncogene partially overlap in antisense transcriptional orientation. This syntenic relationship is conserved in most vertebrates but not in teleost fish, with the exception of the zebrafish, where *mkrn2 *and *raf1 *are closely linked on chromosome 11 (Figure 3). Strikingly, in medaka *raf1 *is located close to *mkrn4 *but not *mkrn2*. We further analyzed syntenic relationships between genomic regions containing *mkrn2 *and *mkrn4 *in human, chicken, medaka and zebrafish genomes (Figure 3). We could identify a set of genes with duplicated copies linked to *mkrn2 *and *mkrn4 *in at least one species (for example *ppard/pparg *in human, chicken and medaka). Other genes like *raf1 *or *cnpb *showed only one copy but either linked to *mkrn2 *in some species or to *mkrn4 *in others, suggesting differential loss of paralogous copies after duplication.

These results indicate that *mkrn2 *and *mkrn4 *are parts of ancient large duplicated regions present in distant vertebrate species. Hence, these paralogons might have been generated by one of the whole genome duplications having occurred in the ancestral vertebrate lineage, the so called 1R and 2R duplications [26-29]. Both *mkrn2 *and *mkrn4 *are called ohnologs (paralogous sequences resulting from whole genome duplications). On the other hand, we can not exclude the occurrence of a very large segmental gene duplication having generated these duplicated blocks.

The region containing *mkrn1 *shows little conservation between mammals and fish (data not shown). We could not find any evidence for synteny between the *mkrn1*-containing region and other *mkrn *regions. It has been previously proposed that *mkrn1 *and *mkrn2 *are indeed the result of a single gene duplication event [17].

### Retroposition and the evolution of the makorin gene family

In addition to *mkrn1*, *mkrn2 *and *mkrn4*, multiple other *mkrn*-like sequences are present in mammalian genomes. Some of these sequences correspond to intronless retrocopies of *mkrn1*. Their functional status concerning expression, regulation and mutual interference has been the source of controversial debate [16,30].

In order to identify all vertebrate *mkrn-*related sequences, we searched over the Ensembl Genome Browser the current genome releases of representatives of all vertebrate groups including all mammalian genomes [24]. The great majority of identified sequences corresponded to retrogenes of *mkrn1 *(Table 1), with the characteristic absence of introns due to reverse transcription of the mature mRNA molecule [31]. Besides *mkrn3*, which probably corresponds to an expressed functional retrogene present in human and mouse, as many as ten different retrogenes located on eight different chromosomes were identified in the human genome. Most of them presented inactivating mutations or severely truncated open reading frames. Almost no EST corresponding to these retrogenes was detected in databases. Hence, most of these *mkrn1 *retrocopies probably correspond to pseudogenes. Six *mkrn1 *retropseudogenes located on five different chromosomes were identified in the mouse.

**Table 1:** Mkrn1- and mkrn2-related retro(pseudo)genes in human and mouse genomes.

Location	Gene names	Annotation	EST support
Human *mkrn1*			

Hsa7 140.152.840-140.179.369	*MKRN1, RNF61, ENSG00000133606*	source protein-coding gene with introns	expressed in many adult tissues

Hsa15 23.810.454-23.813.164	*MKRN3, D15S9, MGC88288, RNF63, ZFP127, ZNF127*	protein-coding retrogene	EST data for blood, brain, esophagus, liver, placenta, prostate and skin

Hsa1 43.356.765-43.358.101	ENSG00000237090, RP11-342M1.6, MKRNP4*	retropseudogene	no EST support

Hsa2 153.707.170-153.707.338	*ENSG00000234932*	retropseudogene	no EST support

Hsa3 130.647.120-130.646.607	located in intron of ATP2C1 (ENSG00000017260), MKRNP5*	retropseudogene	no EST support

Hsa9 99.488.172-99.489.449	*RP11-535M15.2, ENSG00000233820, makorin ring finger protein 1 pseudogene*	retropseudogene	no EST support

Hsa12 88.177.249-88.178.345	*MKRN9P MKRNP6, MKRN5, MKRN9, RNF65, ZNF127L3, ENSG00000213267*	retropseudogene	no EST support

Hsa12 88.176.895-88.176.996	*ENSG00000237340*	retropseudogene	Hypothalamus BI457939.1

Hsa20 45.092.541-45.093.763	*MKRNP3, ENSG00000225849, RP5-981L23.2, DB295917.1*	retropseudogene	no EST support

HsaX 40.693.508-40.697.060	*MKRNP5, MKRN4, ZNF127-Xp, ZNF127L1, ZNF-Xp, ENSG00000238222, OTTHUMG00000024109,*	partially processed retropseudogene	no EST support

HsaX 73.380.895-73.382.363	*MKRNP1, ZNF127-Xq, RP13-204A15.3, ENSG00000224430*	retropseudogene	no EST support

HsaX 91.627.539-91.627.653	inside intron of *PCDH11X*	retro(pseudo?)gene, very short but intact ORF	no EST support

HsaY 5.352.120-5.352.234	inside intron of *PCDH11Y*	retro(pseudo?)gene, very short but intact ORF	no EST support

Mouse *mkrn1*			

Mmu6 39.347.803-39.370.437	*Mkrn1, ENSMUSG00000029922, NM_018810*	source protein-coding gene with introns	widely expressed in adult tissue with strong expression in adult gonad and embryonic nervous system [ref. [30] and EST data]

Mmu7 69.563.293-69.564.927	*Mkrn3, ENSMUSG00000070527*	protein-coding retrogene	EST data for brain, inner ear, pancreas, spinal cord and thymus

Mmu2 27.338.749-27.339.289	no annotation	newly annotated retropseudogene	no EST support

Mmu3 129.843.302-129.845.025	no annotation	retropseudogene	no EST support

Mmu5 89.267.034-89.268.427	*Mkrn1-ps1, AC160533.6, ENSMUSG00000082389, AF494488*	retropseudogene	no EST support

Mmu5 111.334.263-111.334.760	*AC121934.4 (ENSMUSG00000081104), OTTMUSG00000028665*	retropseudogene	no EST support

Mmu13 77.911.041-77.911.846	*in Intron of 1110033M05Rik (ENSMUSG00000064138)*	retropseudogene	no EST support

MmuX 54.923.521-54.923.664	*RP23-435H13.3, OTTMUSG00000017520*	retropseudogene	no EST support

Mouse *mkrn2*			

Mmu6 115.551.979-115.568.688	*Mkrn2, ENSMUSG00000000439, C81377; 2610002L04Rik*	source protein-coding gene with introns	EST data for several tissue

Mmu10 30.093.050-30.095.334	no annotation	retropseudogene	no EST support

MmuX 13.504.413-13.506.637	*BX005215.13, ENSMUSG00000082736, RP23-280F9.1, OTTMUSG00000016911*	Partially processed retropseudogene (contains only 2 introns)	no EST support

Mmu2 106.753.075-106.754.515	no annotation	retropseudogene	no EST support

Positions are based on human GRCh37 and mouse NCBIM37 assemblies. Gene names marked with "*" refer to reference [4].

We detected *makorin3 *and other retrocopies of *makorin1 *only in mammals and not in chicken, frog and all five fish genomes analyzed. An *mkrn3 *sequence was detected in opossum, suggesting an event of retroposition that took place before the split of marsupials from therian mammals. No *mkrn3 *was found in the current genome draft of the platypus.

Retrocopies of *mkrn2 *were found in the mouse but neither in the human nor in non-mammalian vertebrate genomes (Table 1). These three retropseudogenic copies of *mkrn2 *are located on three different chromosomes in *M. musculus*. No retrocopy of *mkrn4 *was found in all vertebrate genomes analysed, but three potentially functional retrocopies of the single intron-containing *mkrn *gene were identified in the fruit fly *Drosophila melanogaster*. Taken together, these observations demonstrate an intriguing tendency of the *mkrn *gene family to generate duplicated copies by retroposition.

### Structural evolutionary dynamics in the Makorin family

**Figure 4:**
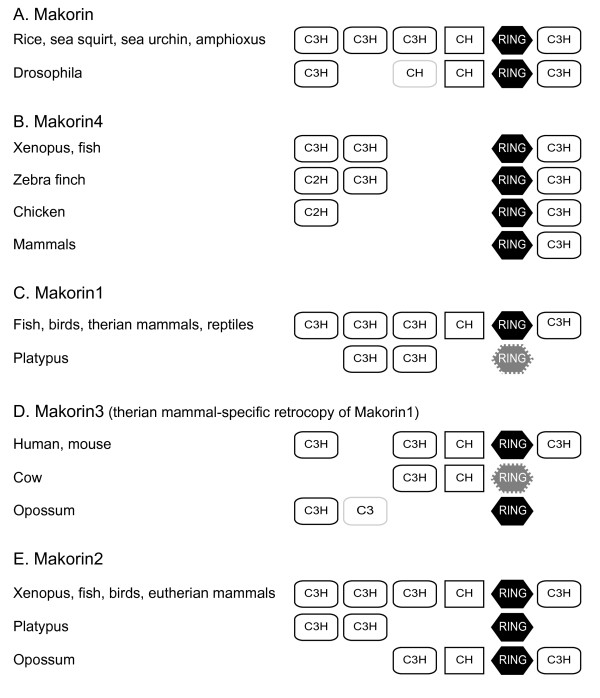
**Zinc finger domains in the Makorin protein family**. Black hexagons indicate the RING domain, white rectangles the C3H zinc fingers. Dashed lines and lighter content indicate incomplete motifs due to sequence changes or loss. The structure of Mkrn2 for *Xenopus *is based on a *X. laevis *EST (EF626804) since the *tropicalis *sequence is incomplete and lacks the RING domain. Accession numbers are provided in Additional file 2.

The evolution of the nature and number of zinc fingers in the Makorin protein family was investigated (Figure 4). In most plants and invertebrate species (but not in *Drosophila*), the single Makorin protein contains the RING domain flanked by three C3H zinc fingers on its N-terminal side and one C3H zinc finger on its C-terminal side. An additional motif rich in cysteine and histidine residues (the CysHis motif) is located between the third C3H zinc finger and the RING domain (Figures 1 and 4). This structure, likely to be ancestral in vertebrates, is also found in most, if not all Mkrn1 and Mkrn2 proteins.

In contrast, this otherwise well conserved structure was not found in Mkrn4 proteins (Figures 1 and 4). While the RING domain and the C-terminal C3H zinc finger are found in all Mkrn4 sequences, the third C3H zinc finger and the CysHis motif are missing and have been probably lost in the ancestral *mkrn4 *gene early after duplication.

Only fish and *Xenopus *have kept the first two C3H complete domains, which are both absent from mammalian sequences. In birds, a remnant of one of these domains has been retained in chicken and zebra finch under the form of a C2H domain, the second C3H domain having been maintained only in zebra finch but not in chicken.

These results indicate that the evolution of the Mkrn4 protein is associated with the dynamic loss of zinc finger domains otherwise conserved in Mkrn proteins. This is consistent with a to some extent faster evolution also reflected by the longer branches in the Makorin-like group in the phylogenetic tree (Figure 2). Another example of zinc finger domain loss is visible in *mkrn3*: this *mkrn1*-derived retrogene encodes a protein having lost the second C3H zinc finger (Figures 1 and 3) in human and mouse.

In the much conserved Mkrn1 and Mkrn2 groups, the platypus sequences present a reduced number of zinc fingers, like the opossum Mkrn2 sequence. This might reflect a biological reality, but might also be an artifact of local poor sequence assembly quality of the genome drafts for these species.

### Evolution of *makorin *expression in vertebrates

**Figure 5:**
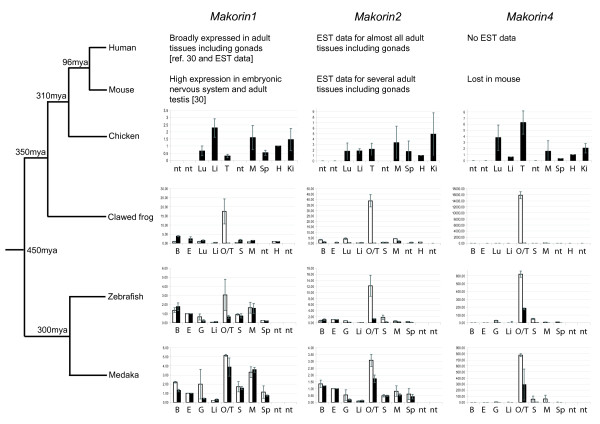
**RT-qPCR expression analysis of *makorin *genes in vertebrate adult tissues**. Q-PCR values for each gene were normalized to expression levels of *rpl7 *using the 2-DDCT method [71]. Expression in eye (fish) or heart (chicken and frog) was set as a reference (value: 1); Data are presented as mean ± standard deviation of two independent quantitative real-time PCR experiments (average of two independent reverse transcription reactions, each tested with two PCR reactions). Black bars represent male tissues and organs, white bars female tissues and organs. B brain, E eye, G gills, H heart, I intestine, K kidneys, Lu lung, Li liver, M muscle, O ovary, S skin, Sp spleen, T testis, nt not tested.

**Figure 6:**
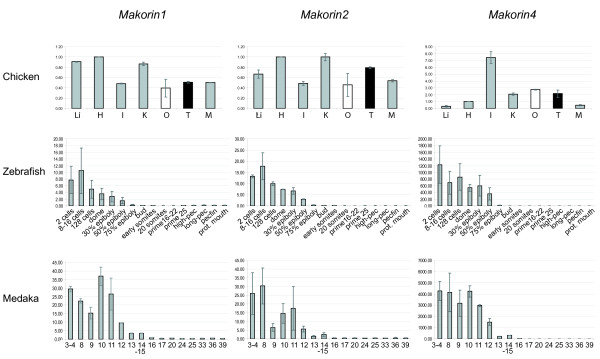
**RT-qPCR expression analysis of *makorin *genes in vertebrate embryos and embryonic tissues**. Q-PCR values for each gene were normalized to expression levels of *rpl7 *using the 2-DDCT method [71]. Expression in adult eye (fish) (Fig. 5) or heart (chicken) was set as a reference (value: 1). Data are presented as mean ± standard deviation of two independent quantitative real-time PCR experiments (average of two independent reverse transcription reactions, each tested with two PCR reactions). Black bars represent male tissues and organs, white bars female tissues and organs. For grey bars, the sex of the donor(s) is undetermined (embryos prior to sexual differentiation) or both sexes have been mixed (frog samples). H heart, I intestine, K kidneys, Li liver, M muscle, O ovary, T testis. Embryonic stages are referred to as [53] for medaka and [54] for zebrafish, respectively.

We compared the expression of the different vertebrate *makorin *genes in different species representative of the fish (medaka and zebrafish), amphibian (the African clawed frog *Xenopus laevis) *and bird (chicken) lineages (published and EST data are generally available for mammals) (Figures 5 and 6). This might allow to assess the functional evolution of *mkrn *genes in vertebrates and to estimate the ancestral expression pattern and function in the primitive vertebrate before gene duplication.

In adult tissue, the whole *mkrn *gene family showed a clear gonad-biased expression in fish and frog, with particular high expression in ovary (Figure 5). Accordingly, *mkrn1 *is upregulated during ovarian maturation in flatfish, with strong expression in previtellogenic ovarian follicles [32]. Such a specificity of expression was not observed in birds and mammals, even if *mkrn1 *has been reported to be strongly expressed in mouse adult testis [4] and *mkrn4 *showed a slightly testis-biased expression in chicken.

Taken together, these results suggested that the ancestral *mkrn *gene was preferentially expressed in the gonads in a primitive vertebrate, with conservation of expression pattern after duplication in fish and amphibians but partial relaxation of this specificity and possible extension of functions in birds and mammals.

*Mkrn *expression during early embryonic stages in fish largely reflected maternally stored mRNA expressed during oogenesis [33,34], since mRNA levels were very high at very early stages (Figure 6). RNA levels then decreased throughout development, probably due to dilution effect through continuous cell division, and reached a very low level shortly after midblastula transition (MBT), the state of zygotic transcription activation. MBT takes place around stage 11 in medaka [35] and starts at cycle 10 corresponding to 512 cell stage in zebrafish [36] (between 128 cell stage and dome in our experiments). Maternal expression was observed for all three *mkrn *genes in both medaka and zebrafish, suggesting here again an ancestral expression pattern. As observed in adults, no tissue-specific expression was observed in chicken embryos one day before hatching (Figure 6).

## Conclusion

Through the identification of *mkrn4*, a new member of the makorin gene family present in tetrapods and ray-finned fishes, we were able to gain some insights into the structural and functional evolution of this E3 ubiquitin ligase gene family. We could show that different evolutionary dynamics affect different *mkrn *genes in vertebrates. While *mkrn1 *and *mkrn2 *were detected in all bony vertebrates studied, *mkrn4 *was found to be absent from rat and mouse, two rodent murids, as well as from rabbit, a lagomorph. In addition, both Mkrn1 and Mkrn2 proteins have retained the ancestral arrangement of zinc fingers, with four C3H motifs surrounding the RING domain responsible for the ubiquitin ligase activity. In contrast, Mkrn4 has lost several motifs early after its formation through duplication, and subsequent lineage-specific zinc finger loss was observed. Taken together, these observations suggest that Mkrn4 is subject to more relaxed evolutionary constraints than Mkrn1 and Mkrn2, with possible development of new general and lineage-specific functions. However, all Mkrn proteins contain the RING domain with adjacent C-terminal C3H zinc finger, indicating that E3 ubiquitin ligase activity and interactions with nucleic acids might be properties common to all Makorin proteins. It appears therefore possible that Mkrn4 function, if still required in rat and mouse, might be performed by Mkrn1 or Mkrn2.

Through comparative genomics, we could find evidence that *mkrn2 *and *mkrn4 *have been duplicated together with other genes through a large-scale duplication. These genes are embedded in paralogons, i.e. in large syntenic chromosomal blocks of duplicated genes within a same genome. These paralogons were found in both tetrapods and ray-finned fish, tracing back their origin to at least 450 million years ago. We propose that *mkrn2 *and *mkrn4 *duplication was the result of either 1R or 2R, the two events of genome duplication having taken place at the basis of the vertebrate lineage [12,26-28]. Both 1R and 2R arose before the split between cartilaginous and bony fish; one might therefore expect the presence of both *mkrn2 *and *mkrn4 *in the genome of elephant shark. However, only *mkrn2 *was detected. This might be explained by secondary loss of *mkrn4 *or incomplete coverage of the elephant shark genome. The molecular mechanism of formation of *mkrn1 *remains elusive. Involvement of 1R or 2R appears possible (but we could not find any evidence for that) or of a more local event of segmental duplication. Conservation of intron position between *mkrn1*, *mkrn2 *and *mkrn4 *(data not shown) suggests that retroposition was not involved.

After formation of *mkrn1*, *mkrn2 *and *mkrn4*, the *makorin *gene family was shaped by lineage-specific retroposition-mediated duplications in vertebrates. Such retrogenes are formed by reverse transcription of generally mature mRNA molecules followed by integration of the formed cDNA into a new location of the genome. In mammals, retrogene formation is catalyzed by reverse transcriptase encoded by LINE-1 retrotransposons [37]. Retrogenes were detected for *mkrn1 *and *mkrn2 *in mammals but not in other vertebrates, and not for *mkrn4*. Most of the retrogenes are pseudogenes, but at least one of them, *mkrn3*, is functional and conserved in therian mammals. Hence, retroposition is an evolutionary mechanism of duplication and functional diversification in the *mkrn *family. There is at the moment no satisfying explanation for the strong tendency of the *mkrn *family to generate retrogenes. Germ cell expression, necessary for retrogene formation and transmission to the next generation, might predispose *mkrn *genes to duplication through retroposition. Goncalves *et al. *[38] have proposed that the gene expression level in the germ line is correlated with the number of retropseudogenes, since abundant mRNAs are more likely to be taken as a substrate by reverse-transcriptases. However, high *mkrn *expression is not the only explanation, since no retrocopy was detected for *mkrn *genes in amphibians and fish, in which they show a strong gonadal expression. Other factors might be involved, for example the affinity of expressed reverse transcriptases for mRNA molecules.

Finally, comparative expression analysis showed a strongly gonad-biased expression pattern for *mkrn1*, *mkrn2 *and *mkrn4 *in medaka and zebrafish (ray-finned fishes) and amphibians, with particularly strong expression in ovary. Even if some *mkrn *genes showed also preferential gonadal expression in chicken and mouse, expression was much less specific in birds and mammals. Maternal RNA was detected for all three genes in early medaka and zebrafish embryos. Taken together, these results suggest that the ancestral single-copy *mkrn *gene was gonad-specific in primitive vertebrates, with a possible role in sexual development or gonad functions. This ancestral gene was also possibly maternally expressed and therefore potentially involved in fundamental processes such as fertilization, cell division or germ cell development [34].

Nothing is known concerning the functions of Mkrn proteins in fish and amphibian gonads and embryos, with the exception of a described role for Mkrn2 as a negative regulator of neurogenesis in *Xenopus *[18]. At the moment it is therefore difficult to propose a function for the ancestral vertebrate Mkrn protein, but results obtained for Mkrn1 suggest a link with the regulation of cell proliferation and apoptosis [4,6]. An involvement of the ubiquitin pathway in testis and spermatogenesis has been proposed for different organisms and testis-specific transcripts of different ubiquitin-interacting factors have been reported (for example [39-43]). Some data indicated an involvement of the ubiquitin pathway in the maintenance and development of ovarian germ cells in the fly [44] and the zebrafish [45] and during maturation of the ovary in mouse [46]. Several examples of maternally-expressed RING finger proteins with essential functions during development have been reported [47-51]. Multispecies comparative functional analysis of *mkrn *genes is now required to better understand the evolution of this gene family in vertebrates.

## Methods

### Fish

Platyfish (*Xiphophorus maculatus*, population Rio Jamapa WLC1274, closed colony stock derived from Jp163A), zebrafish (*Danio rerio*, strain AB/TU) and medaka (*Oryzias latipes*, strain Hd-rR) were kept under standard conditions at the PRECI aquarium facility of the IFR128 Biosciences Gerland-Lyon Sud (Lyon, France). Fish embryos were raised to the required stages of development in E3 embryo media [52] at 26°C for medaka and 28.5°C for zebrafish. Medaka and zebrafish embryos were staged according to Iwamatsu [53] and Kimmel *et al. *[54], respectively. Chicken samples (embryonic chicken, one day before hatching and male adult tissue) were provided by B. Pain (Institut de Génomique Fonctionnelle de Lyon, ENS de Lyon, France). Adult *Xenopus laevis *samples were gifts from F. Vollmar (Department of Cell and Developmental Biology, University of Würzburg, Germany) and C. Schultheis (Physiological Chemistry I, University of Würzburg, Germany). Experimental research reported here has been performed following internationally recognized guidelines (DDSV, Direction Départementale des Services Vétérinaires de Lyon, agreement number A 69 387 0602).

### *In silico *analyses

Protein and nucleotide sequences of *mkrn *genes were identified using BLAST/BLAT searches against GenBank [55], the current Ensembl genome assemblies ([24] version 57, March 2010) of human (*Homo sapiens*), chimpanzee (*Pan troglodytes*), gorilla (*Gorilla gorilla*), orangutan (*Pongo pygmaeus*), macaque (*Macaca mulatta*), mouse lemur (*Microcebus murinus*), mouse (*Mus musculus*), guinea pig (*Cavia porcellus*), horse (*Equus caballus*), rabbit (*Oryctolagus cuniculus*), cow (*Bos taurus*), opossum (*Monodelphis domestica*), wallaby (*Macropus eugenii*), platypus (*Ornithorhynchus anatinus*), chicken (*Gallus gallus*), clawed frog (*Xenopus tropicalis*), zebrafish (*Danio rerio*), medaka (*Oryzias latipes*), green spotted pufferfish (*Tetraodon nigroviridis*), fugu (*Takifugu rubripes*), three-spined stickleback (*Gasterosteus aculeatus*), sea squirts (*Ciona intestinalis *and *Ciona savignyi*), the amphioxus genome (*Branchiostoma floridae*) [56] and the elephant shark genome (*Callorhinchus milii*) [57]. Automatic genome annotation was refined manually using EST data and related protein sequences with the help of FGENESH [58].

Nucleotide and protein sequences were loaded into BioEdit [59] and aligned using MUSCLE [60,61] and CLUSTALW2 [62]. Alignments were checked manually and ambiguously aligned regions were removed before phylogenetic analysis. Phylogenetic trees were calculated based on a 131 amino acid alignment including the RING domain and the downstream adjacent C3H zinc finger. Maximum likelihood reconstruction was done using PhyML 3.0 [63,64], with 1000 bootstrap replicates under the LG model. Similar results were obtained using the neighbor-joining method as implemented in MEGA4 [65] with bootstrap values of 10.000 replicates. MEGA4 was also used to visualize phylogenetic trees. To establish syntenic relationships between genomes we used Genomicus [66], Synteny Database [67] and information provided by Ensembl Gene Trees [24]. Annotation of conserved protein motifs was performed with Motif Scan [68] and NCBI conserved domains [12].

Ka/Ks calculation was peformed using KaKsCalculator under the MYN model [69] on pairwise nucleotide alignments done with LASTZ [70] and MUSCLE [60,61]. Indicated Ka/Ks values are averages of values obtained for all possible pairwise comparisons.

### RT-qPCR expression analysis

Total RNA was extracted from separated male and female tissues (brain, eyes, gills, heart, intestine, kidneys, lung, liver, muscle, ovary, skin, spleen and testis) and from whole embryos using the TRIReagent (Molecular Research Center, Inc.). After DNase treatment, reverse transcription was performed starting from 1 μg of total RNA using the RevertAid First Strand Synthesis kit (Fermentas) and random hexamer primers. Real-time PCR was done on 2 μl of dilutional series of cDNA starting from a dilution of factor 20 using IQTM Custom SYBR^® ^Green Supermix (Bio-Rad). PCR amplification was monitored with a CFX96 RealtimeSystem (Bio-Rad). After one incubation step at 95°C for 3 min, the thermal cycling protocol was as follows: 95°C for 10 seconds, 55-62°C (primer-dependent annealing temperature) for 20 seconds and 72°C for 15 seconds during 40 cycles. Primers were designed manually and ordered from Invitrogen (Additional file 3). For each primer set, the efficiency of the PCR reaction was measured in duplicate on serial dilutions (factor 10) of the cDNA sample. Real-time PCR efficiencies (*E*) for each reaction were calculated from the slope of the standard curve using the equation E = 10^(-1/slope)^-1 as implemented in the BioRad CFX manager V1.5 software, with 100% efficiency as an indicator of a robust assay. All results reported here are averages of two independent reverse transcription reactions, which have been each tested with two PCR reactions. For quantification, data were analyzed by the 2-DDCT method [71], by normalizing to the housekeeping gene *rpl7 *(ribosomal protein l7). Expression was normalized to eye tissue (fish) or heart (due to lack of eye samples for female frog and chicken).

## Authors' contributions

AB carried out the molecular genetic studies. AB and JNV performed sequence analysis. AB, HDC and JFB constructed the platyfish cDNA library. AB and AD identified and characterized *mkrn4 *in platyfish. AB, DGA and JNV designed the study and wrote the paper. All authors read and approved the final manuscript.

## Supplementary Material

Additional file 1 **Makorin sequence accession numbers**. Click here for file

Additional file 2 **Genes used for synteny analysis**. Click here for file

Additional file 3 **Real-time qPCR primers used**. Click here for file
