# Supplementary material for: The vertebrate makorin ubiquitin ligase gene family has been shaped by large-scale duplication and retroposition from an ancestral gonad-specific, maternal-effect gene
Source: BMC Genomics. 2010 Dec 20;11:721. doi: 10.1186/1471-2164-11-721 (PMC3022923; doi:10.1186/1471-2164-11-721)
Supplement: Additional file 1 — Makorin sequence accession numbers. [file 1471-2164-11-721-S1.DOC]

**Additional file 1**

**Makorin sequence accession numbers.**

Accession numbers of makorin sequences used for molecular phylogeny (Fig. 2) and zinc finger analysis (Fig. 4)

| **species** | **accession number** |
| --- | --- |
| **Makorin1** |  |
| Chicken | ENSGALP00000020932 |
| Cow | ENSBTAP00000010932 |
| Elephant shark | AAVX01046064.1 |
| Takifugu | ENSTRUP00000030501 |
| Green spotted pufferfish | ENSTNIP00000003623 |
| Human | ENSP00000255977 |
| Medaka | ENSORLP00000015988 |
| Mouse | ENSMUSP00000031985 |
| Opossum | ENSMODP00000017114 |
| Platypus | ENSOANP00000010963 |
| Threespined stickleback | ENSGACP00000025415 |
| Zebrafish | ENSDARP00000061069 |
| Western clawed frog | ENSXETP00000005731 |
|  | |
| **Makorin2** |  |
| African clawed frog | EF626804 |
| Cow | ENSBTAP00000005927 |
| Chicken | ENSGALP00000007982 |
| Elephant shark | AAVX01102336.1 |
| Takifugu | ENSTRUP00000007391 |
| Green spotted pufferfish | ENSTNIP00000007547 |
| Human | ENSP00000170447 |
| Medaka | ENSORLP00000012328 |
| Mouse | ENSMUSP00000000449 |
| Opossum | ENSMODP00000007792 |
| Platypus | ENSOANP00000023532 |
| Three-spined stickleback | ENSGACP00000004448 |
| Western clawed frog | ENSXETP00000043492 |
| Zebrafish | ENSDARP00000016210 |
|  | |
| **Makorin3** |  |
| Cow | ENSBTAP00000048771 |
| Human | ENSP00000313881 |
| Mouse | ENSMUSP00000091898 |
| Opossum | ENSMODG00000022796 |
|  |  |
| **Makorin4** | |
| Antarctic toothfish | overlapping ESTs FE218499 and FE230161 |
| Atlantic cod | CO541599 |
| Channel catfish | overlapping ESTs FD350543 and CK419269 |
| Chicken | ENSGALP00000034421 |
| Common roach | EG540007 |
| Cow | XP_001788373 |
| Fathead minnow | overlapping ESTs DT177973, DT289118 and DT091762 |
| Green spotted pufferfish | ENSTNIP00000002237, manually three exons added 5’ through sequence comparison with other fish sequences |
| Guppy | overlapping ESTs ES386309 and ES380911 |
| Horse | XP_001494443 |
| Human | EAX03829.1 |
| Medaka | ENSORLP00000008302, manually corrected |
| Opossum | XP_001378442 |
| Platypus | ENSOANP00000027603 |
| Three-spined stickleback | ENSGACP00000010952, two exons 5’ manually added, one 3’ through sequence comparison with fish sequences |
| Western clawed frog | ENSXETP00000033120 |
| Zebra finch | XP_002196697.1 |
| Zebrafish | ENSDARP00000095121 |
|  |  |
| **Makorin** | |
| Amphioxus | jgi|Brafl1|185653|gw.29.157.1 |
| Fruit fly | FBpp0078038 |
| Rice | BAD61603.1| |
| Sea squirts | ENSCINP00000014748, ENSCSAVP00000010889 |
| Sea urchin | XP_783167.2 |
