# Supplementary material for: The vertebrate makorin ubiquitin ligase gene family has been shaped by large-scale duplication and retroposition from an ancestral gonad-specific, maternal-effect gene
Source: BMC Genomics. 2010 Dec 20;11:721. doi: 10.1186/1471-2164-11-721 (PMC3022923; doi:10.1186/1471-2164-11-721)
Supplement: Additional file 2 — Genes used for synteny analysis. [file 1471-2164-11-721-S2.DOC]

**Additional file 2**

**Genes used for synteny analysis.**

Ensembl accession numbers of genes used for synteny analysis in Fig. 3. Accession numbers correspond to Ensembl gene entries, except for *mkrn4* in human, which is a GenBank entry. For the newly identified gene *lhfpl5b* in chicken, chromosomal location is given based on the current Ensembl genome release ([24] May, 2010).

| **Zebrafish** |  |
| --- | --- |
| *brpf1* | ENSDARG00000046136 |
| *brpf3a* | ENSDARG00000062958 |
| *brpf3b* | ENSDARG00000074238 |
| *cnbp* | ENSDARG00000070922 |
| *fam107a* | ENSDARG00000059399 |
| *fam107b* | ENSDARG00000079869 |
| *foxp4* | ENSDARG00000076120 |
| *itpr1a* | ENSDARG00000074149 |
| *itpr3a* | ENSDARG00000076582 |
| *itpr3b* | ENSDARG00000061741 |
| *lhfpl5a* | ENSDARG00000045023 |
| *lhfpl5b* | ENSDARG00000056458 |
| *mkrn2* | ENSDARG00000007630 |
| *mkrn4* | ENSDARG00000028295 |
| *ppardb* | ENSDARG00000009473 |
| *raf1a* | ENSDARG00000031638 |
| *raf1b* | ENSDARG00000059406 |
| **Medaka** |  |
| *brpf1* | ENSORLG00000011135 |
| *brpf3a* | ENSORLG00000000358 |
| *brpf3b* | ENSORLG00000003896 |
| *cnbp* | ENSORLG00000011453 |
| *fam107a* | ENSORLG00000009794 |
| *foxp1a* | ENSORLG00000003081 |
| *foxp1b* | ENSORLG00000013419 |
| *foxp4* | ENSORLG00000015849 |
| *ip6k2a* | ENSORLG00000001247 |
| *ip6k2b* | ENSORLG00000016832 |
| *itpr1a* | ENSORLG00000012718 |
| *itpr1b* | ENSORLG00000014557 |
| *lhfpl5a* | ENSORLG00000016106 |
| *lhfpl5b* | ENSORLG00000006380 |
| *mkrn2* | ENSORLG00000009824 |
| *mkrn4* | ENSORLG00000006615 |
| *pparg* | ENSORLG00000004432 |
| *ppard* | ENSORLG00000006636, |
| *raf1* | ENSORLG00000011471 |
| **Human** |  |
| *BRPF1* | ENSG00000156983 |
| *BRPF3* | ENSG00000096070 |
| *Cnbp* | ENSG00000169714 |
| *FAM107A* | ENSG00000168309 |
| *Foxp1* | ENSG00000114861 |
| *Foxp4* | ENSG00000137166 |
| *IP6K1* | ENSG00000176095 |
| *IP6K2* | ENSG00000068745 |
| *IP6K3* | ENSG00000161896 |
| *ITPR1* | ENSG00000150995 |
| *ITPR3* | ENSG00000096433 |
| *Lhfpl5* | ENSG00000197753 |
| *Mkrn2* | ENSG00000075975 |
| *Mkrn4* | EAX03829.1 |
| *Ppard* | ENSG00000112033 |
| *Pparg* | ENSG00000132170 |
| *Raf1* | ENSG00000132155 |
| **Chicken** |  |
| *brpf1* | ENSGALG00000006640 |
| *brpf3* | ENSGALG00000000776 |
| *cnbp* | ENSGALG00000005001 |
| *fam107a* | ENSGALG00000007139 |
| *foxp1* | ENSGALG00000007769 |
| *foxp4* | ENSGALG00000003375 |
| *lhfpl5* | ENSGALG00000000897 |
| *lhfpl5b* | newly annotated Gga12, bp 11.724.727-11.725.164 |
| *ip6k1* | ENSGALG00000002510 |
| *ip6k2* | ENSGALG00000005701 |
| *ip6k3* | ENSGAL00000002881 |
| *itpr1* | ENSGALG00000008294 |
| *itpr3* | ENSGALG00000003149 |
| *lhfpl5* | ENSGALG00000000897 |
| *mapk14a* | ENSGALG00000019759 |
| *mkrn2* | ENSGALG00000004991 |
| *mkrn4* | ENSGALG00000002568, |
| *ppard* | ENSGALG00000002588 |
| *pparg* | ENSGALG00000004974 |
| *raf1* | ENSGALG00000004998 |
