# Supplementary material for: The vertebrate makorin ubiquitin ligase gene family has been shaped by large-scale duplication and retroposition from an ancestral gonad-specific, maternal-effect gene
Source: BMC Genomics. 2010 Dec 20;11:721. doi: 10.1186/1471-2164-11-721 (PMC3022923; doi:10.1186/1471-2164-11-721)
Supplement: Additional file 3 — Real-time qPCR primers used. [file 1471-2164-11-721-S3.DOC]

**Additional file 3**

**Real-time qPCR primers used.**

Real-time qPCR primers for the amplification of *mkrn1*, *mkrn2*, *mkrn4* and the control gene *rpl7* in zebrafish (*Danio rerio*), medaka (*Oryzias latipes*), chicken (*Gallus gallus*) and frog (*Xenopus laevis*). F, forward; R, reverse.

| Primer | Sequence 5'-3' |
| --- | --- |
| *rpl7* |  |
| Zebrafish-F | GCCAGATCTTCAACGGTGTCT |
| Zebrafish-R | AGGCAATGTAGGGCTCTGCAA |
| Medaka-F | CGCCAGATCTTCAACGGTGTAT |
| Medaka-R | GGCTCAGCAATCCTCAGCAT |
| Chicken-F | CTACAAGCGTGGTTATGGCA |
| Chicken-R | CATGCAGATGATGCCAAGTT |
| Frog-F | GAACCAGCGTATCCCTCTGA |
| Frog-R | GCCAACGGTGTAGACTTCAT |
|  |  |
| *mkrn1* |  |
| Zebrafish-F | GCCACTGCTACTGCCTCAAG |
| Zebrafish-R | CCTTGTCCTCCACCCAGTATT |
| Medaka-F | AACTTTGTGATCCCAAGCGAGTA |
| Medaka-R | GGCGTGCTTGTAGAAGCAGTT |
| Chicken-F | CAAACACGCGTATCCTGATG |
| Chicken-R | ACCATTCTCTCGCTCCTCAA |
| Frog-F | AAACAACTGTGCCCGTACGC |
| Frog-R | TGCAGTTGGACAAGATGCCG |
|  |  |
| *mkrn2* |  |
| Zebrafish-F | TGTCCAGAGTGTCGTGTGG |
| Zebrafish-R | GTTCCTCTGCCCTGGTCAA |
| Medaka-F | ATATGCCGCAAATGGACATT |
| Medaka-R | CAGCCTCAAATGCAAGGAGAC |
| Chicken-F | GTTCGTCATTCCCAGTGCTT |
| Chicken-R | CCTCCAAATGGACAAGTTCCT |
| Frog-F | AGGCCAAGCCCCATTCATAC |
| Frog-R | CTCACCAGCCTGGGCAAAAG |
|  |  |
| *mkrn4* |  |
| Zebrafish-F | AATGGATCGTGTCGATATGGT |
| Zebrafish-R | CTGCTCACCGAAGAGCCTTC |
| Medaka-F | TGCATCTACCGTCACGAATG |
| Medaka-R | TCCATGTTGGGCAGAAGTACA |
| Chicken-F | CTTCCCAAGCACAGTCATCA |
| Chicken-R | TGAGCTGCTGCTTCTCATCT |
| Frog-F | TATGAACGTAGTCGGGATGT |
| Frog-R | AACCTCATTCTGGAAGTCTC |
